# The Antioxidant and Anti-Inflammatory Properties of *Merremia umbellata* Extract

**DOI:** 10.3390/antiox12122037

**Published:** 2023-11-23

**Authors:** Sunny Chung Lee, Jongmin Ahn, Jina Kim, Joo-Yeon Lee, Juhae Kim, Md. Salah Uddin, Sang Woo Lee, Choon Young Kim

**Affiliations:** 1 Department of Molecular and Medical Pharmacology, University of California, Los Angeles, CA 90095-1735, USA; chung_lee@alumni.brown.edu; 2 Natural Product Central Bank & Natural Medicine Research Center, Korea Research Institute of Bioscience and Biotechnology (KRIBB), Cheongju 28116, Chungbuk, Republic of Korea; jmahn@kribb.re.kr; 3 Department of Food and Nutrition, Yeungnam University, Gyeongsan 38541, Gyeongbuk, Republic of Korea; jinakim@ynu.ac.kr (J.K.); jooyeonlee@ynu.ac.kr (J.-Y.L.); 4 Research Institute of Human Ecology, Yeungnam University, Gyeongsan 38541, Gyeongbuk, Republic of Korea; kimjh825@yu.ac.kr; 5 Ethnobotanical Database of Bangladesh, Tejgaon, Dhaka 1208, Bangladesh; sohailmsu@knu.ac.kr; 6 International Biological Material Research Center, Korea Research Institute of Bioscience and Biotechnology, Daejeon 34141, Republic of Korea; ethnolee@kribb.re.kr

**Keywords:** *Merremia umbellata* Hallier f., antioxidant activity, anti-inflammatory activity, UHPLC-PDA-QTOF, TLR4/NF-κB pathway

## Abstract

*Merremia umbellata* Hallier f. (MU) has been used as an anti-inflammatory agent to treat burns and scales. However, the potential anti-inflammatory mechanisms of action of this plant have not been elucidated. This study aimed to assess the antioxidant and anti-inflammatory effects of the leaf and shoot of MU grown in Bangladesh. The MU extract exhibited antioxidant activities as demonstrated by DPPH and ABTS free-radical-scavenging activities and the total polyphenol and total flavonoid contents. MU extract significantly reduced the lipopolysaccharide (LPS)-stimulated nitric oxide (NO) production in RAW264.7 macrophage. Accordingly, the gene levels of inducible NO synthase and cyclooxygenase-2 were suppressed. The MU extract alleviated the LPS-induced expression of TLR4, NF-κB, and inflammatory cytokines (*TNF-α*, *IL-6*, and *IL-1β*). The constituents of a MU extract were tentatively identified using UHPLC-PDA-QTOF/MS techniques. The main compounds were identified as 3,4-dicaffeoylquinic acid, 3,5-dicaffeoylquinic acid, quercitrin, and 4,5-dicaffeoylquinic acid. Molecular docking analysis revealed that these compounds interact with TLR4 protein, with quercitrin showing the highest binding affinity among them. Overall, our findings demonstrate the antioxidant and in vitro anti-inflammatory activities of MU and its potential compounds to target the TLR4-NF-κB signaling pathway. These findings are potentially used to further explore promising natural food ingredients that are effective in regulating inflammation.

## 1. Introduction

*Merremia umbellata* Hallier f. (MU), commonly known as hogvine or yellow merremia and also called morning glory, belongs to the family of Convolvulaceae [1]. MU is a perennial climbing vine that can grow up to 3 m tall. Notably, the leaves of MU are narrow, broadly ovoid, and approximately 10 cm long. MU is widely grown in the humid tropical regions of America, Africa, and South and Southeast Asia [2]. MU leaves have been used in folk remedies to treat wounds, sores, burns, rheumatism, and other inflammatory conditions [3,4,5]. To date, most studies on MU have been conducted on MU grown in regions of America, such as the northern coast of Colombia; these studies have revealed that the leaves of MU have antibacterial, antifungal, anti-inflammatory, and anti-oxidant properties [6,7,8]. Previous studies have also provided phenomenological evidence of the health-promoting actions of MU but not its potential underlying cellular mechanisms. To gain a deeper understanding of the biological activity of MU, it is essential to have a comprehensive grasp of its qualitative composition. Some studies have conducted phytochemical screening of MU to explore its biological activities, including antioxidant and antibacterial properties, revealing the presence of specific compounds such as polyphenols, terpenoids, and steroids. However, detailed information about the specific groups and quantities of those chemical compounds is lacking. Presently, there is a limitation in conducting a thorough phytochemical analysis and determining the qualitative composition of MU.

Inflammation is the first line of defense against pathogens and injuries [9]. However, a dysregulated inflammatory response may lead to inflammation-associated diseases, including cardiovascular diseases, obesity, and type 2 diabetes [10]. Macrophages are crucial innate immune cells, whose activation status plays a role in the induction and resolution of inflammation [11,12]. During the inflammatory response, macrophages activate inflammatory signaling cascades and release pro-inflammatory mediators and cytokines, including nitric oxide (NO), tumor necrosis factor-alpha (TNF-α), interleukin-6 (IL-6), and interleukin-1 beta (IL-1β) [10,13].

The inflammatory signaling cascade begins with the binding of the inflammatory stimuli, such as inflammatory cytokines or bacterial lipopolysaccharide (LPS), to a specific receptor, such as Toll-like receptors (TLRs). Notably, the TLR4-nuclear transcription factor-κB (NF-κB) pathway is the most well-known cascade [9]. Activation of NF-κB involves the inhibitor of κB (IκB) phosphorylation, resulting in the translocation of NF-κB into the nucleus. Activated NF-κB, mediated using TLR4/myeloid differentiation factor-88 signaling in the upstream region, induces the gene expression of the pro-inflammatory cytokines mentioned earlier. The increased expression levels of nitric oxide synthase 2 (NOS2) and cyclooxygenase-2 (COX-2), which mediate the inflammatory response by increasing the production of NO and prostaglandin 2, respectively, are well-known downstream effects of NF-κB activation [14,15].

LPS triggers macrophage activation and initiates an inflammatory response [16]. Thus, the LPS-stimulated macrophage model has been widely used to assess the anti-inflammatory activity and mechanism of action of potential medications, including plant extracts. In this study, we evaluated the antioxidant properties of the MU extract by assessing its radical-scavenging activity and antioxidant content, such as polyphenols including flavonoids. Thereafter, we used LPS-stimulated RAW264.7 macrophages to investigate the anti-inflammatory effect of the MU extract and the underlying molecular mechanisms, with a focus on TLR4-NF-κB signaling.

## 2. Materials and Methods

### 2.1. Chemical Reagents

Phosphate-buffered saline (PBS) and 3-(3,4-dimethyl-thiazolyl-2)-2,5-diphenyl tetrazolium bromide (MTT) were purchased from Welgene (Daegu, Republic of Korea) and DUCHEFA (Haarlem, The Netherlands), respectively. The primers were purchased from Bioneer (Daejeon, Republic of Korea). Power SYBR™ Green PCR Master Mix and Dulbecco’s modified Eagle medium (DMEM) were obtained from Thermo Fisher Scientific (Waltham, Finland). The iScript cDNA synthesis kit was purchased from Bio-Rad Laboratories Inc. (Hercules, CA, USA) and fetal bovine serum (FBS) was purchased from GIBCO (Grand Island, NY, USA). Griess reagent, dimethyl sulfoxide (DMSO), and other reagents were purchased from Sigma-Aldrich (St. Louis, MO, USA).

### 2.2. Plant Collection, Voucher Specimen Information, and Extract Preparation Method

*Merremia umbellata* (L.) Hallier f. (MU) was collected in Kachhapia (21.449893, 92.203380), Garjania, Cox’s Bazar district, Chittagong division, Bangladesh, and identified by Md. Salah Uddin at Ethnobotanical Database of Bangladesh in May 2014. A voucher specimen (accession number, KRIB 0054951) of the retained material is preserved at the herbarium of KRIBB. The dried leaves and shoots of MU (74 g) were extracted with 1 L of 99.9% (*v*/*v*) methanol using repeated sonication (15 min) and resting (2 h) for 3 days at 45 °C. The resultant product was filtered with non-fluorescence cottons, and concentrated using a rotary evaporator (N-1000SWD, EYELA CO., Tokyo, Japan) under reduced pressure at 45 °C. Finally, a total of 4.07 g of methanol extract of MU was obtained by freeze-drying. The extraction efficacy was 5.5%. Freeze-dried MU extract was dissolved in DMSO for further studies.

### 2.3. Determination of the Total Phenolic and Total Flavonoid Contents

The total phenolic content (TPC) was analyzed using the Folin–Ciocalteu method [17]. Briefly, the diluted MU leaf extract was mixed with Folin–Ciocalteu reagent (10%, *v*/*v*). After 5 min, 700 mM sodium carbonate solution was added, and the absorbance was measured at 765 nm following incubation for 1 h at room temperature (RT). The TPC was calculated using a standard curve obtained using gallic acid as the standard material. The results are presented as gallic acid equivalents in milligrams per gram of extract (mg GAE/g of extract). The total flavonoid content (TFC) was measured as previously described [18]. The diluted MU extract was mixed with a sodium nitrite solution and allowed to react for 5 min. Aluminum chloride hexahydrate (10%) was added, and the reaction was allowed to proceed for 5 min; thereafter, 1 M sodium hydroxide was added. The absorbance was measured at 510 nm. Catechin was used as a standard, and the results are presented as catechin equivalents in milligrams per gram of extract (mg CE/g of extract).

### 2.4. Determination of the Antioxidant Activity

The antioxidant activity of the MU extract was evaluated using 1,1-diphenyl-β-picrylhydrazyl (DPPH) and 2,2-azino-bis (3-ethylbenzothiazoline-6-sulfonic acid (ABTS) radical-scavenging assays. Following dilution of the MU extract to different concentrations, a 0.02% DPPH solution was added, and the solutions were incubated at RT for 30 min. The absorbance was measured at 510 nm. ABTS (7.4 mM) and potassium persulfate (2.6 mM) were prepared and mixed in a 1:1 ratio. The mixture was stored in the dark at RT overnight. After dilution of the ABTS^•+^ solution with distilled water to obtain an absorbance of 0.70 ± 0.02 units at 734 nm, the diluted MU and the reagent were mixed at a ratio of 1:19. Following incubation for 6 min at RT, the absorbance was measured at 732 nm. DPPH radical-scavenging activity of ascorbic acid (AA) was used to extrapolate to determine the AA equivalent (AAE) values, while a Trolox calibration curve of ABTS radical-scavenging activity was utilized to calculate the Trolox equivalent (TE) value.

### 2.5. Cell Culture and Treatment

The murine macrophage RAW264.7 cell line was purchased from the American Type Culture Collection (Manassas, VA, USA) and maintained in DMEM supplemented with 10% FBS, 100 µg/mL of streptomycin, and 100 U/mL of penicillin at 37 °C with 5% CO_2_. The medium was replaced every three days. Upon reaching 80% confluence, the cells were subcultured or treated with MU in the presence of 200 ng/mL of LPS for 24 h. The no treatment (NT) group was administered 1 μg/mL of DMSO, a solvent for MU.

### 2.6. MTT Assay

Cell viability was assessed using the MTT assay. The cells were seeded in a 96-well plate at a density of 1 × 10^5^ cells/well overnight, and then treated with 200 ng/mL of LPS and different concentrations (0, 12.5, 25, 50, or 100 μg/mL) of MU. At 24 h after treatment, the cell culture medium was aspirated, and 10% FBS–DMEM with 5 mg/mL MTT solution was added to each well and incubated at 37 °C for 1 h. The supernatant was then aspirated, and the purple product, formazan converted from tetrazolium salts by the viable cells and was dissolved with DMSO. Finally, the absorbance of the product was measured at 570 nm using a microplate reader (BioTek, Inc., Winooski, VT, USA), and cell viability was quantified.

### 2.7. Nitric Oxide Assay

The anti-inflammatory activity was assessed by measuring NO production in the culture medium. The cells were seeded in a 96-well plate at a density of 1 × 10^5^ cells/well overnight, and then treated with or without LPS and different concentrations of MU (12.5, 25, 50, or 100 μg/mL). At 24 h after treatment, equal volumes of medium (culture supernatant) and Griess reagent were mixed and incubated for 10 min at RT in the dark. The absorbance was measured at 540 nm using a microplate reader (BioTek, Inc.). The NaNO_2_ standard curve was plotted as a function of NO production and subsequently used to determine the level of NO production, expressed µM of NaNO_2_.

### 2.8. RNA Isolation, cDNA Synthesis, and Quantitative RT-PCR Analysis

The cells were cultured in 24-well plates at 5 × 10^5^ cells/well for 24 h and then treated with or without LPS and different concentrations of MU (25, 50, or 100 μg/mL) for 24 h. After treatment, the cells were washed with ice-cold PBS, and total RNA was isolated using TRIzol reagent (15596018, Invitrogen Life Technologies, Carlsbad, CA, USA), according to the manufacturers’ protocol. The resulting RNA pellet was dissolved in DEPC water, and its purity and concentration were assessed using a NanoDrop spectrophotometer (Thermo Fisher Scientific, Waltham, MA, USA). For each sample, cDNA was synthesized from 1 μg of total RNA using the iScript Synthesis Kit (Bio-Rad Laboratories, Inc., Hercules, CA, USA) and a SimpliAmp Thermal Cycler instrument (Applied Biosystems, Waltham, MA, USA). Quantitative real-time PCR analysis was performed using Power SYBR™ Green PCR Master Mix on a StepOne Plus Real-Time PCR System (Applied Biosystems). The results were normalized to those of the endogenous control, β-actin, and relative gene expression levels were analyzed using the 2^−ΔΔCt^ method. The primer sequences are listed in Table 1.

### 2.9. Protein Extraction and Western Blot Analysis

The cells were cultured in a 6-well-plate at 5 × 10^5^ cells/well and treated with or without LPS and different concentrations of MU (25, 50, or 100 μg/mL) for 24 h. After treatment, the cells were washed with ice-cold PBS and lysed with cell lysis buffer (100 mM Tris-HCl, 100 mM NaCl, 0.5% Triton-X, 1 mM sodium orthovanadate, and 10 mM sodium fluoride) containing a protease-inhibitor cocktail (GenDEPOT, Barker, TX, USA). Protein lysates were obtained from the supernatants via centrifugation at 15,900× *g* for 15 min. The protein concentration of each lysate was determined using the Bradford method and a Bio-Rad protein assay (Bio-Rad Laboratories, Inc.). Equal amounts of proteins were denatured, separated on sodium dodecyl sulfate-polyacrylamide gels, and transferred onto PVDF membranes. The membranes were blocked with 5% nonfat milk in Tris-buffered saline with Tween 20 (TBST) for 1 h and incubated overnight with antibodies against β-actin, TLR-4, NF-κB, and IL-1β at 4 °C. After washing with TBST buffer, a horseradish peroxidase-conjugated secondary antibody (Jackson ImmunoResearch, West Grove, PA, USA) was added to the membrane at a 1:2000 dilution. After washing with TBST buffer, the membrane was developed using ECL Prime Western Blotting Detection Reagent (GE Healthcare, Milwaukee, WI, USA) on an X-ray film. ImageJ software (ImageJ bundled with 64-bit Java 8, National Institutes of Health, Bethesda, MA, USA) was used to determine the band densities of proteins relative to those of β-actin.

### 2.10. UHPLC-PDA-QTOF/MS Analysis

The analysis of the MU extract was conducted using an ACQUITY UPLC I-Class PLUS System (Waters Co., Milford, MA, USA) equipped with an Acquity PDA and a XEVO-G2XS QTOF/MS spectrometer. Separation was accomplished using an ACQUITY BEH C18 column (2.1 × 100 mm, 1.7 μm, Waters Co.) with elution solvents consisting of purified water obtained from a Milli-Q Academic water purification system (Merck Millipore, Darmstadt, Germany), acetonitrile (Merck Millipore), and formic acid (Sigma-Aldrich). The mobile phase consisted of A (0.1% formic acid in water) and B (0.1% formic acid in acetonitrile), following a gradient profile: 5% B from 0.0 to 1.0 min; 5–100% B from 1.0 to 20.0 min; 100% B from 20.0 to 22.3 min; and 5% B from 22.4 to 25.0 min. A flow rate of 0.4 mL/min was consistently kept, and a column temperature of 35 °C was held steady throughout the analysis. The MS parameters included a source temperature of 110 °C, desolvation temperature of 350 °C, capillary voltage of 0.3 kV, and cone voltage of 40 V. Leucine-enkephalin was used as the reference lock mass in both the negative mode [*m*/*z* 554.2615] and positive mode [*m*/*z* 556.2771], and data were collected in the MSe mode within the m/z range of 100 to 1000, employing collision energies of 6 eV and 25–50 eV. Tentative identification was conducted based on spectroscopic analysis, and some phenolic compounds were compared with reference standards and in-house library.

### 2.11. Quantitative Analysis of Major Components

The calibration curve was constructed by plotting the integrated peak areas corresponding to the concentration of each standard. These curves were then used to calculate the regression equation, correlation coefficient, as well as the Limit of Detection (LOD), and Limit of Quantification (LOQ). LOD and LOQ were determined by calculating them as 3.3 times and 10 times, respectively, the standard deviation of the blank divided by the slope of the calibration curve [19].

### 2.12. Molecular Docking Analysis

The TLR4 protein structures were downloaded from Protein Data Bank (PDB code: 3UL7) in the PDB format and were prepared by adding polar hydrogen atoms, and Kollman charges before docking were processed in MGLTools. MGLTools was used to convert them into the pre-requisite PDBQT format. The AutoDock Tools version 1.5.6 software was used to perform docking for the major components of MU extract into the active sites of TLR4 receptor proteins. The size and dimension of the Grid box were selected using co-crystallized ligand coordinates within the target protein.

### 2.13. Statistical Analysis

The results are expressed as the mean ± standard error of the mean (SEM) of triplicate measurements. Statistical analyses were performed using Duncan’s multiple range test with SPSS software (version 25.0; IBM, Chicago, IL, USA). Statistical significance was set at *p* < 0.05.

## 3. Results

### 3.1. Antioxidant Properties of the MU Extract

**Figure 1:**
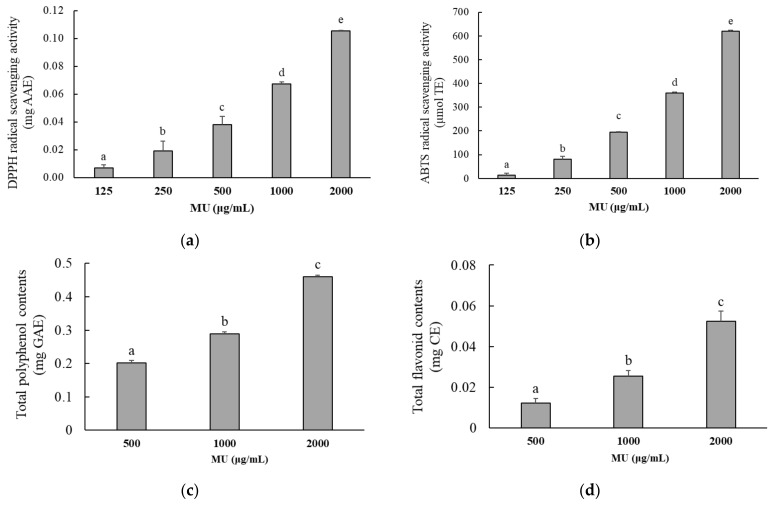
Antioxidant activity, total polyphenol content, and the flavonoid contents of *Merremia umbellata* (MU) extract. (**a**) DPPH radical-scavenging activity is expressed in mg ascorbic acid equivalent (mg AAE). (**b**) ABTS radical-scavenging activity is expressed in µmol trolox equivalent (µmol TE). (**c**) Total polyphenol content is expressed in mg gallic acid equivalent of MU extract (mg GAE). (**d**) Total flavonoid content is expressed in mg catechin equivalent of MU extract (mg CE). Different letters indicate significant difference with one-way ANOVA followed by Duncan’s multiple range test (*p* < 0.05).

The antioxidant activity of MU extract was measured using the DPPH and ABTS radical-scavenging assays. As shown in Figure 1a,b, the free radical-scavenging activity of MU extract ranged from 125 to 2000 μg/mL, and the activity increased as the concentration of MU extract increased. At 2000 μg/mL, the DPPH radical-scavenging activity of MU extract was 0.11 ± 0.01 mg AAE, and the ABTS radical-scavenging activity of MU was 619.0 ± 4.48 µmol TE. The total polyphenol and flavonoid contents were assessed, as shown in Figure 1c,d. These contents were equivalent to 0.461 ± 0.004 mg GAE for TPC and 0.052 ± 0.005 mg CE for TFC. As shown in Table 2, a high correlation was found between radical-scavenging activity, TPC, and TFC (*p* < 0.001).

### 3.2. Effect of the MU Extract on Cytotoxicity in LPS-Stimulated RAW264.7 Macrophages

**Figure 2:**
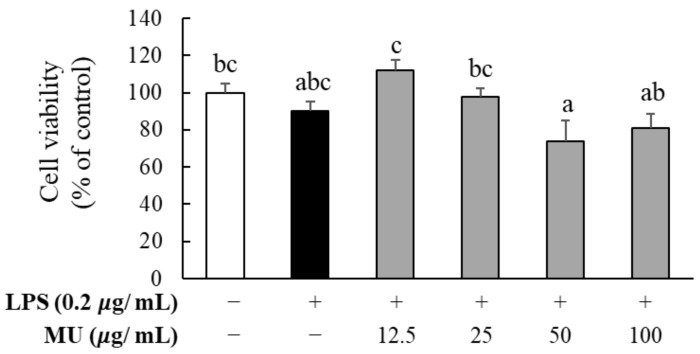
Effect of *Merremia umbellata* (MU) on the cell viability of LPS-induced RAW264.7 macrophages. The cell viability at 24 h after co-treatment with different concentrations (25, 50, and 100 μg/mL) of MU, and 2 μg/mL of LPS was measured using the MTT assay. Different letters indicate significant difference with one-way ANOVA followed by Duncan’s multiple range test (*p* < 0.05).

In order to determine the cytotoxicity of RAW264.7 macrophages using MU extract in the presence of LPS, the cells were incubated with 0.2 μg/mL of LPS and various concentrations (0, 12.5, 25, 50, and 100 μg/mL) of MU extract for 24 h. As shown in Figure 2, the MTT assay revealed that LPS treatment lowered cell viability; however, the difference was not statistically significant. Treatment with MU extract in the presence of LPS also affected cell viability; however, no significant differences were observed. Therefore, subsequent experiments were performed using concentrations up to 100 μg/mL of MU extract, which did not induce significant cytotoxicity.

### 3.3. Effect of MU Extract on LPS-Induced NO Production and Pro-Inflammatory Enzyme Expression in RAW264.7 Macrophages

**Figure 3:**
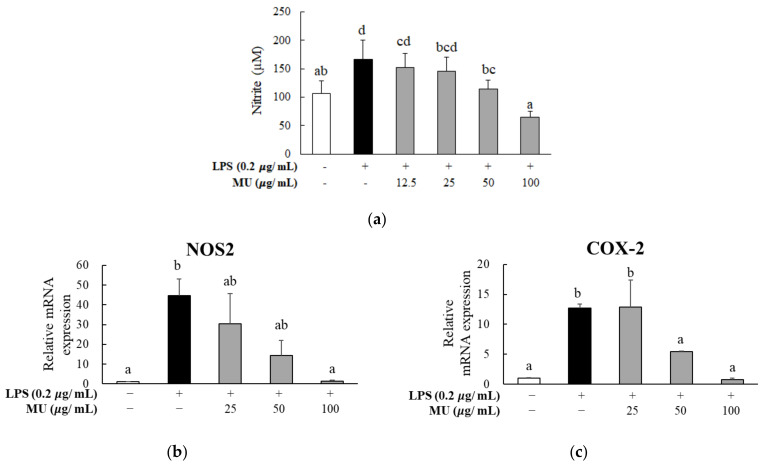
Effect of *Merremia umbellata* (MU) on nitric oxide (NO) secretion and pro-inflammatory-enzyme gene-expression level in LPS-induced RAW264.7 macrophages. After cells were co-treated with different concentrations (25, 50, and 100 μg/mL) of MU and 2 μg/mL of lipopolysaccharide (LPS) for 24 h, (**a**) NO secretion and the gene expression levels of (**b**) nitric oxide synthase 2 (NOS2) and (**c**) cyclooxygenase-2 (COX-2) were determined. Different letters indicate significant difference with one-way ANOVA followed by Duncan’s multiple range test (*p* < 0.05).

The anti-inflammatory properties of MU extract were investigated by assessing the NO levels produced in RAW264.7 macrophages upon stimulation with LPS. As shown in Figure 3a, LPS-only treatment induced a significant increase (1.57-fold) in NO production compared with no treatment group. When 12.5 to 100 μg/mL of MU extract was added, LPS-induced NO production was suppressed in a dose-dependent manner (0.10-, 1.14-, 1.46-, and 2.60-fold reduction, respectively). The gene expression of the pro-inflammatory enzymes was examined with 25 to 100 μg/mL of the MU extract. The MU extract was found to suppress the mRNA expression of NOS2 and COX-2 in LPS-treated RAW264.7 macrophages (Figure 3b,c). In cells subjected to LPS-only treatment, 100 and 50 μg/mL of the MU extract significantly suppressed the gene expression of NOS2 and COX-2, respectively.

### 3.4. Effect of MU Extract on LPS-Induced TLR4/NF-κB Signaling Activation in RAW264.7 Macrophages

**Figure 4:**
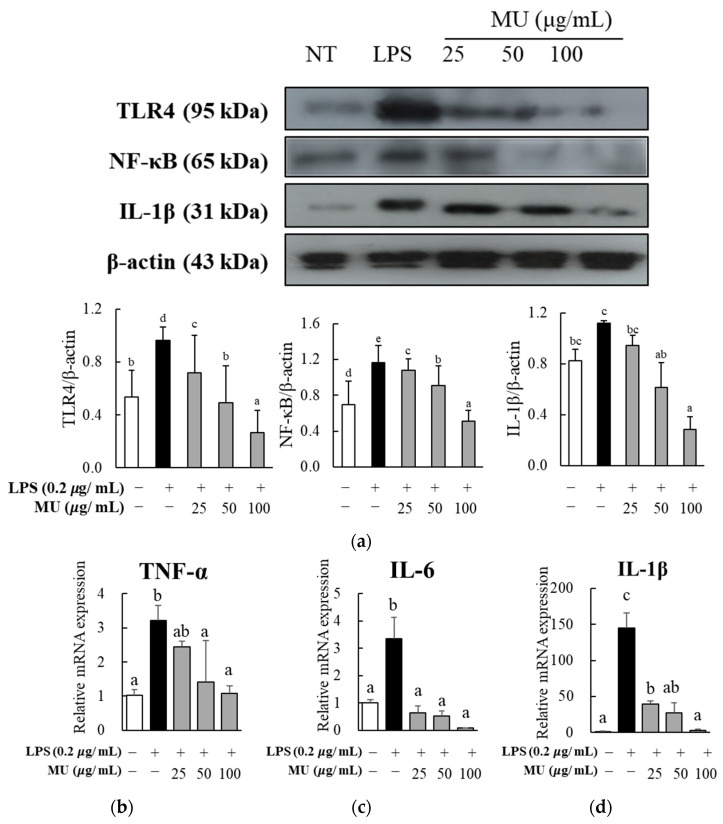
Effect of *Merremia umbellata* (MU) on the activated TLR4-NF-kβ pathway in LPS-induced RAW264.7 macrophages. After cells were co-treated with different concentrations (25, 50, and 100 μg/mL) of MU and 2 μg/mL of lipopolysaccharide (LPS) for 24 h, (**a**) the protein expression levels of Toll-like receptor 4 (TLR-4), nuclear factor-kappa B (NF-κB), and interleukin-1 beta (IL-1β), and the gene expression levels of tumor necrosis factor-α (**b**) (TNF-α), (**c**) interleukin-6 (IL-6), and (**d**) interleukin-1 beta (IL-1β) were measured. Different letters indicate significant difference with one-way ANOVA followed by Duncan’s multiple range test (*p* < 0.05).

Since the reduction in NO production and pro-inflammatory enzyme gene expression supported the anti-inflammatory action of MU extract in LPS-treated RAW264.7 macrophages, we opted to examine the expression of genes and proteins involved in the inflammatory TLR4/NF-κB signaling pathway. The LPS-stimulated induction of TLR4 and NF-κB protein expression was suppressed by MU extract in a dose-dependent manner. The highest dose (100 μg/mL) of MU extract decreased the protein expression of TLR4 by 90.5% and that of NF-κB by 84.5%. The protein expression of IL-1β, one of the downstream inflammatory cytokines of the TLR4/NF-κB signaling pathway, was also reduced using MU treatment (Figure 4a). Analysis of the mRNA expression of not only IL-1β, whose protein expression was assessed, but also other downstream inflammatory cytokines, including TNF-α and IL-6, revealed dose-dependent reduction owing to treatment with MU extract. The highest dose (100 μg/mL) of MU extract inhibited the expression of TNF-α, IL-6, and IL-1β by 66.2%, 97.5%, and 98.1%, respectively, compared to treatment with LPS alone (Figure 4b).

### 3.5. Constituents of MU Extract

**Figure 5:**
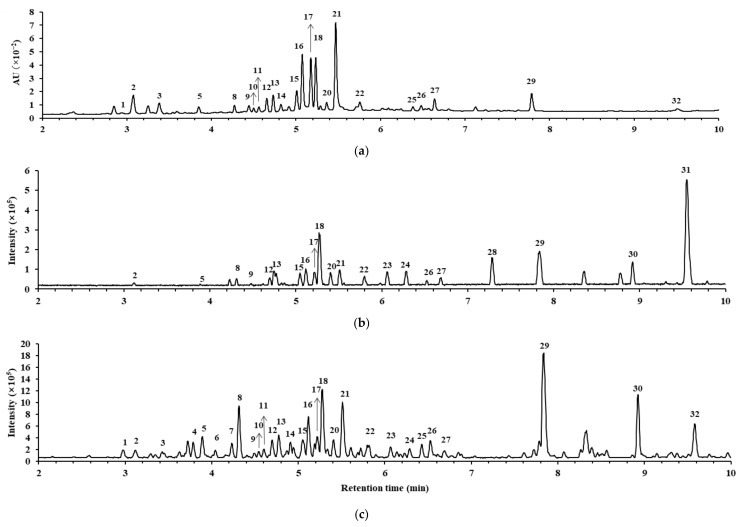
The chromatograms of *Merremia Umbellata* (MU) extract. (**a**) UV chromatogram at 254 nm. (**b**) MS chromatogram in positive ion mode. (**c**) MS chromatogram in negative ion mode.

In order to figure out the chemical composition and potential bioactive constituents, the phytoconstituents of the MU extract were analyzed using UHPLC-PDA-QTOF/MS (Figure 5). The tentative identification of constituents was conducted by analyzing UV and MS spectral data, revealing that the predominant classes of components included flavonoid glycosides and phenolic compounds (Table 3). Notably, the main constituents were identified to be 3,4-dicaffeoylquinic acid (**16**), 3,5-dicaffeoylquinic acid (**17**), quercitrin (**18**), and 4,5-dicaffeoylquinic acid (**21**) according to the UV chromatogram at 254 nm. The quantitative determination results for the key components in MU extract are presented in Table 4. The amounts of quercitrin, 3,4-dicaffeoylquinic acid, 3,5-dicaffeoylquinic acid, and 4,5-dicaffeoylqunic acid in MU were 7.89, 4.91, 4.29, and 2.66 mg/g, respectively.

### 3.6. Identification of TLR4 and Compounds from MU Extract

**Figure 6:**
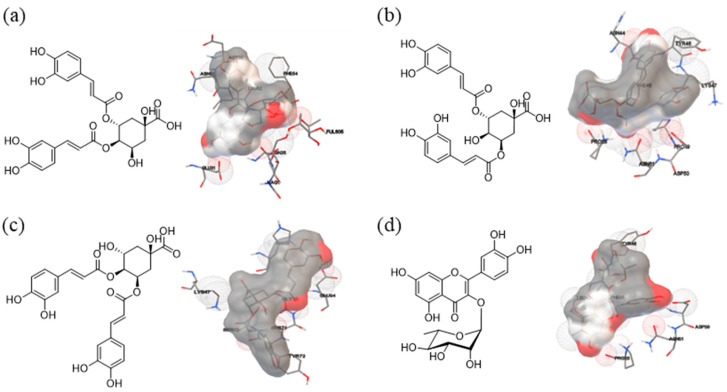
Optional conformational structure of interactions between main compounds found in MU extract with the TLR4 receptor. Chemical structures of (**a**) 3,4-dicaffeoylquinic acid, (**b**) 3,5-dicaffeoylquinic acid, (**c**) 4,5-dicaffeoylquinic acid, and (**d**) quercitrin compounds are placed on the left side, while 3D structures of each compound interacting with the active site of the TLR4 receptor protein are shown on the right side.

Toll-like receptor (TLR) 4, a receptor for LPS, plays an important role in the initiation of inflammation response through the activation of the NF-κB pathway [21]. In order to identify the molecular targets of a compound from the MU extract involved in the inhibition of TLR4, we performed molecular docking analysis using AutoDock Tools 1.5.6 software. The active sites of TLR4 protein were docked with 3,4-dicaffeoylquinic acid, 3,5-dicaffeoylquinic acid, quercitrin, and 4,5-dicaffeoylquinic acid (Figure 6a–d). The molecular docking energies are showed in kcal/mol and are presented in Table 5. The 3,4-dicaffeoylquinic acid exhibited eight active sites: ASN44, TYR46, PHE45, LYS47, PRO28, PRO49, ASN51, and ASP50. The docking bind energy was −3.65 kcal/mol. The 3,5-dicaffeoylquinic acid bound with TLR4 via seven active sites: LYS47, HIS68, GLU94, GLY70, SER71, SER73, and TYR72. It was discovered that the molecular-binding energy score was −3.78 kcal/mol. The quercitrin showed six active sites: TYR46, LEU43, PHE45, ASP50, ASN51, and PRO28. The binding score was −5.19 kcal/mol. The binding of TLR4 to 4,5-dicaffeoylquinic acid was discovered to involve nine active sites, PRO28, CYS29, ASN51, ASP50, LEU52, PHE54, PRO53, NAG1, and FUL805, and docking energy is −4.68 kcal/mol. The quercitrin exhibited the highest binding score among the five compounds identified in MU extract. Upon comparing the calculated molecular docking energy levels for the three isomers of dicaffeoylquinic acid, it becomes evident that the position of the caffeoyl group significantly influences the interaction with the TLR-4 receptor. Notably, the binding scores follow this order: 5-position > 4-position > 3-position of caffeoyl group on quinic acid.

## 4. Discussion

Inflammation is triggered by harmful stimuli such as pathogens and toxic materials and is associated with the pathogenesis of a several chronic diseases. Several anti-inflammatory agents have been developed to inhibit the overproduction of cytokines and mediators. Well-known anti-inflammatory drugs include non-steroidal anti-inflammatory drugs (NSAIDs), such as ibuprofen and aspirin. As these commonly used drugs have side effects, such as gastrointestinal and cardiovascular adverse events [22], safer and more effective anti-inflammatory agents from natural substances, such as botanical sources, have been evaluated. Therefore, we propose MU as a potential natural ingredient for relieving pro-inflammatory responses based on its anti-oxidative properties. Moreover, we firstly reported qualitative composition of MU. At concentrations up to 100 μg/mL, the MU extract significantly suppressed the expression of pro-inflammatory mediators and cytokines in an LPS-stimulated macrophage culture system. Our data suggest that TLR4/NF-κB signaling is the main pathway for the anti-inflammatory action of the MU extract.

The present data have elucidated the antioxidant activity of MU through analyses of the DPPH, ABTS, TPC, and TFC contents. The DPPH and ABTS radical-scavenging inhibitory activities were determined to be 85.2% and 75.5%, respectively, corresponding to IC_50_ values of 815.2 μg/mL and 1226.2 μg/mL, respectively. Notably, these IC_50_ values markedly exceeded those reported in previous studies. For instance, Castro Guerrero et al. reported IC_50_ values of 140.7 and 199.5 μg/mL for DPPH and ABTS radical-scavenging activities, respectively [6]. The disparity in these values can be attributed to the geographical origin of the MU samples; the MU used in this study was collected from Bangladesh, in contrast to the Colombian origin of the MU in the study of Castro Guerrero et al. Additionally, biological properties of MU were found to be influenced by the specific plant part used. Specifically, the leaves and shoots of MU exhibited TPC and TFC values of 54.3 mg GAE/g of extract and 25.8 mg CE/g of extract, respectively, while the stems of MU collected from the Khulna University campus in Bangladesh displayed a TPC of 57.1 mg GAE/g of extract and a TFC of 65.1 mg QE/g of extract [23].

Moreover, the MU extract was subjected to UHPLC-PDA-QTOF/MS analysis, revealing phytoconstituents of the MU extract and the qualitative data of key components (16, 17, 18, and 21) included 3,4-dicaffeoylquinic acid, 3,5-dicaffeoylquinic acid, quercitrin, and 4,5-dicaffeoylquinic acid. While previous phytochemical screening assay merely confirmed the presence of certain chemical compounds, including polyphenols, terpenoids, and sterols, the exact groups of bioactive components remained elusive [7]. In this study, we have provided a detailed qualitative analysis of these key compounds. Specifically, the predominant components of MU were found in the following descending order: quercitrin, 3,4-dicaffeoylquinic acid, 3,5-dicaffeoylquinic acid, and 4,5-dicaffeoylqunic acid. This comprehensive identification of bioactive components significantly enriches the understanding of MU’s phytochemical profile, enhancing our knowledge of its potential health-promoting properties.

To the best of our knowledge, this study represents the pioneering investigation into the anti-inflammatory properties and associated mechanisms of MU sourced from Bangladesh. A previous study utilizing MU collected from Colombia had initially reported a reduction in nitric oxide (NO) levels following treatment with MU leaf extract at concentrations ranging from 25 to 100 μg/mL [6]. In the current study, we assessed the anti-inflammatory potential of MU derived from Bangladesh using both the NO assay and gene expression analysis. Our findings demonstrated a dose-dependent reduction in NO release in lipopolysaccharide (LPS)-stimulated RAW264.7 macrophages, consistent with the diminished expression of inflammatory mediators (NOS2 and COX-2) as well as cytokines (IL-1β, TNF-α, and IL-6) due to MU treatment. Comparable outcomes in terms of decreased expression of pro-inflammatory mediators have been reported in LPS-stimulated macrophages treated with plant extracts from the same botanical family, such as *Convolvulus arvensis* L. [24]. Furthermore, other *Convolvulus* plants, including *Convolvulus pluricaulis* Choisy [25] and *Convolvulus fatmensis* [26], have demonstrated anti-inflammatory effects in diverse models, such as adipocyte cell systems and carrageenan-induced rat paw edema models.

Our study has provided valuable insights into the molecular mechanisms underlying the anti-inflammatory effects of MU, specifically focusing on the inhibition of Toll-like receptor 4/nuclear factor-kappa B (TLR4/NF-κB) signaling. We observed a dose-dependent reduction in the protein expression levels of TLR4 and NF-κB in LPS-stimulated macrophages treated with MU. To gain a deeper understanding of the potential components responsible for mediating these inflammatory signaling pathways, we conducted molecular docking analyses. These analyses revealed the active sites of the TLR4 protein interacting with key MU constituents, including 3,4-dicaffeoylquinic acid, 3,5-dicaffeoylquinic acid, quercitrin, and 4,5-dicaffeoylquinic acid. Among these compounds, quercitrin exhibited the highest binding affinity. Previous studies have supported our findings, demonstrating that quercitrin treatment effectively suppressed LPS-induced inflammatory response both in vitro [27] and in vivo [28]. Additionally, quercitrin has been reported to attenuate the NF-κB signaling pathway [29]. This study provides novel insights into potential therapeutic targets by uncovering MU’s ability to inhibit TLR4/NF-κB signaling, an aspect not previously reported within the *Convolvulus* plant family.

## 5. Conclusions

Based on our findings, MU exerts antioxidant activity by scavenging free radicals owing to its total phenolic and flavonoid contents. Major components of MU were flavonoid glycosides and phenolic compounds including 3,4-dicaffeoylquinic acid, 3,5-dicaffeoylquinic acid, quercitrin, and 4,5-dicaffeoylquinic acid. Using the LPS-induced RAW264.7 cell model, we demonstrated that MU may exhibit its inflammatory action via the TLR-NF-κB pathway. Accordingly, our study provides experimental evidence that MU can be developed as a new functional food ingredient to protect against inflammatory diseases.

## Figures and Tables

**Table 1:** Primer sequences used for quantitative real-time PCR.

Gene	Sequence (5′→3′)
*β*-actin	F ^1^: AGA TGA CCC AGA TCA TGT TTG AGA
	R ^2^: CAC AGC CTG GAT GGC TAC GT
COX-2	F: CAT ACT CAA GCA GGA GCA TCC
	R: ACC GCT CAG GTG TTG CAC GTA GTC
IL-1β	F: GCA ACT GTT CCT GAA CTC AAC T
	R: ATC TTT TGG GGT CCG TCA ACT
IL-6	F: TCG GAG GCT TAA TTA CAC ATG TTC
	R: TGC CAT TGC ACA ACT CTT TTC T
NOS2	F: CAG CTG GGC TGT ACA AAC CTT
	R: CAT TGG AAG TGA AGC GTT TCG
TNF-α	F: TGG CCT CCC TCT CAT CAG TT
	R: CAG GCT TGT CAC TCG AAT TTT G
TLR4	F: GCA GAA AAT GCC AGG ATG ATG
	R: AAC TAC CTC TAT GCA GGG ATT CAA G

^1^ F, forward, ^2^ R, reverse.

**Table 2:** Pearson’s correlation coefficient (r^2^) of the relationship between antioxidant activity, total phenolic content (TPC), and total flavonoid content (TFC) of the *Merremia umbellata* extract.

	DPPH (% Inhibition)	ABTS (% Inhibition)	TPC (mg GAE ^c^)	TFC (μg CE ^d^)
DPPH ^a^	1	0.982 **	0.979 **	0.953 **
ABTS ^b^		1	0.969 **	0.931 **
TPC			1	0.979 **
TFC				1

^a^ DPPH, 1,1-diphenyl-β-picrylhydrazyl radical-scavenging activity, ^b^ ABTS, 2,2-azino-bis (3-ethylbenzothiazoline-6-sulfonic acid radical-scavenging activity, ^c^ GAE, gallic acid equivalent, ^d^ CE, catechin equivalent. ** Significant at *p* < 0.01.

**Table 3:** Tentative identification of *Merremia umbellata* components.

No.	tR, min	Molecular Formula	UV	Tentative Identification	Class	Negative Mode				Positive Mode			
						Detected Ion [M–H]^−^	Calculated Ion [M–H]^−^	Error (ppm)	Major Fragment Ions	Detected Ion [M+H]^+^	Calculated Ion [M+H]^+^	Error (ppm)	Major Fragment Ions
**1**	2.98	C_18_H_24_O_12_	266, 271, 277	Unknown	Unknown	431.1189	431.1190	−0.7	327, 165	455.1133	–	–	_
**2**	3.12	C_16_H_18_O_9_	326	Chlorogenic acid ^a^	Phenolic acid	353.0877	353.0873	1.1	191	355.1019	355.1029	−2.8	163
**3**	3.43	C_9_H_8_O_4_	323	Caffeic acid ^a^	Phenolic acid	179.0344	179.0344	0.0	135	–	–	–	–
**4**	3.79	C_18_H_28_O_9_	–	Unknown	Miscellaneous	387.1664	387.1655	2.3	–	–	–	–	–
**5**	3.89	C_19_H_30_O_8_	240	Roseoside ^a^	Miscellaneous	431.1925 [M+HCOO]^−^	431.1917 [M+HCOO]^−^	1.9	281, 153	387.2002	387.2019	−4.4	207
**6**	4.04	C_15_H_20_O_7_	–	Unknown	Unknown	311.1136	311.1131	1.6	–	–	–	–	–
**7**	4.23	C_12_H_18_O_4_	219, 310	Unknown	Unknown	225.1131	225.1127	1.8	–	227.1279	227.1283	−1.8	209
**8**	4.31	C_14_H_24_O_17_	280, 284	Unknown	Unknown	463.0916	463.0935	−4.1	–	–	–	–	–
**9**	4.49	C_17_H_24_O_10_	219	Unknown	Unknown	387.1296	387.1291	1.3	361, 300	–	–	–	–
**10**	4.54	C_27_H_30_O_16_	255, 353	Quercetin–*O*–dihexoside	Flavonoid glycoside	609.1464	609.1456	1.3	300, 271	611.1594	611.1612	−2.9	303
**11**	4.60	C_27_H_30_O_16_	255, 352	Rutin ^a^	Flavonoid glycoside	609.1458	609.1456	0.3	300, 271	611.1612	611.1612	0.0	465, 303
**12**	4.70	C_21_H_20_O_12_	260, 349	Hyperoside ^a^	Flavonoid glycoside	463.0873	463.0877	−0.9	300, 271, 255	465.1029	465.1033	−0.9	303
**13**	4.77	C_21_H_20_O_12_	254, 350	Isoquercitrin ^a^	Flavonoid glycoside	463.0872	463.0877	−1.1	300, 271, 255	465.103	465.1033	−0.6	303
**14**	4.87	C_21_H_20_O_11_	268, 347	Luteolin–*O*–hexoside	Flavonoid glycoside	447.0929	447.0927	0.4	285	449.107	449.1084	−3.1	287
**15**	5.05	C_20_H_18_O_11_	270, 342	Quercetin–*O*–pentoside	Flavonoid glycoside	433.0772	433.0771	0.2	300, 271, 255	435.0919	435.0927	−1.8	303
**16**	5.12	C_25_H_24_O_12_	325	3,4–Dicaffeoylquinic acid ^a^	Phenolic acid	515.1191	515.1190	0.2	353, 191	517.1343	517.1346	−0.6	197, 163
**17**	5.22	C_25_H_24_O_12_	327	3,5–Dicaffeoylquinic acid ^a^	Phenolic acid	515.1185	515.1165	3.9	353, 191	517.1335	517.1346	−2.1	499, 163
**18**	5.28	C_21_H_20_O_11_	255, 345	Quercitrin^a^	Flavonoid glycoside	447.093	447.0927	0.7	300, 271, 255	449.1086	449.1084	0.4	303
**19**	5.34	C_22_H_22_O_12_	267, 336	Isorhamnetin–*O*–hexoside	Flavonoid glycoside	477.1033	477.1033	0.0	314, 299, 271	479.117	497.119	−4.2	317, 303
**20**	5.41	C_20_H_18_O_10_	265, 346	Kaempferol–*O*–pentoside	Flavonoid glycoside	417.0823	417.0822	0.2	284, 255, 227	419.0963	419.0978	−3.6	287
**21**	5.51	C_25_H_24_O_12_	327	4,5–Dicaffeoylquinic acid ^a^	Phenolic acid	515.119	515.1190	0.0	353, 191	517.1345	517.1346	−0.2	327
**22**	5.80	C_21_H_20_O_10_	263, 344	Afzelin ^a^	Flavonoid glycoside	431.0973	431.0978	−1.2	285, 255, 227	433.1144	433.1135	2.1	287
**23**	6.07	C_17_H_17_NO_3_	287, 312	Paprazine [20]	Tyramine (Alkaloid)	282.1133	282.1130	1.1	161, 119	284.1289	284.1287	0.7	147
**24**	6.28	C_18_H_19_NO_4_	285, 318	Feruloyltyramine	Tyramine (Alkaloid)	312.1244	312.1236	2.6	178	314.1392	314.1392	0.0	177
**25**	6.43	C_23_H_24_O_12_	271, 330	Unknown	Flavonoid glycoside	491.1188	491.1190	−0.4	329, 271	493.1334	493.1346	−2.4	270, 242
**26**	6.53	C_30_H_26_O_13_	266, 314	Tiliroside	Flavonoid glycoside	593.1301	593.1295	1.0	447, 284	595.1445	595.1452	−1.2	287
**27**	6.69	C_34_H_30_O_15_	330	3,4,5–tricaffeoylquinic acid ^a^	Phenolic acid	677.1519	677.1506	1.9	515, 439, 315	679.1646	679.1663	−2.5	317
**28**	7.28	C_16_H_17_NO_2_	275	Unknown	Alkaloid	254.1185	254.1181	1.6	161	256.1338	256.1338	0.0	–
**29**	7.84	C_18_H_16_O_10_S	272, 333	Unknown	Flavonoid sulfate	423.0385	423.0386	−0.2	343, 328, 313, 298, 285, 270	425.054	425.0542	−0.5	345, 331
**30**	8.92	C_42_H_66_O_14_	–	Unknown	Triterpenoid saponin	793.4399	793.4374	3.2	631	–	–	–	–
**31**	9.55	C_19_H_18_O_6_	–	Unknown	Unknown	–	–	–	–	343.1189	343.1182	2.0	313, 287
**32**	9.58	C_16_H_14_O_5_	288, 326	Unknown	Flavonoid phytoalexin	285.0769	285.0763	2.1	164	287.092	287.0919	0.3	153

^a^ Identified based on comparison with reference standards or in-house library.

**Table 4:** Quantitative analysis, linear equations, LODs, and LOQs of four main compounds in *Merremia umbellata* extract.

No.	Compound Name	Contents in MU Extract ^a^ (mg g^−1^)	Linear Equations ^b^	Correlation Coefficient (r^2^)	LOD ^c^ (ng mL^−1^)	LOQ ^c^ (ng mL^−1^)
**16**	3,4-Dicaffeoylquinic acid	4.91	y = 6100.3x − 1185.4	0.9999	134	406
**17**	3,5-Dicaffeoylquinic acid	4.29	y = 7585.3x + 87.11	0.9999	164	498
**18**	Quercitrin	7.89	y = 7297.1x − 1050.2	0.9999	87	262
**21**	4,5-Dicaffeoylquinic acid	2.66	y = 15177x − 2183.9	0.9999	119	360

^a^ “Contents in MU extract” refers to the contents of each analyte in the dried MU extract (mg g^−1^). ^b^ y, peak area; x, concentration (µg mL^−1^). ^c^ LOD, Limit of Detection; LOQ, Limit of Quantification.

**Table 5:** Docking scores of four compounds identified from *Merremia umbellata* extract against TLR4 receptor.

Binding Ligand	Amino Acid Involved Interaction	Binding Score (kcal/mol)
3,4-Dicaffeoylquinic acid	ASN44, TYR46, PHE45, LYS47, PRO28, PRO49, ASN51, ASP50	−3.65
3,5-Dicaffeoylquinic acid	LYS47, HIS68, GLU94, GLY70, SER71, SER73, TYR72	−3.78
Quercitrin	TYR46, LEU43, PHE45, ASP50, ASN51, PRO28	−5.19
4,5-Dicaffeoylquinic acid	PRO28, CYS29, ASN51, ASP50, LEU52, PHE54, PRO53, NAG1, FUL805	−4.68

## Data Availability

The data presented in this study are available on request from the corresponding author.
